# Initial screening positivity for suspected scoliosis and machine learning-assisted risk prioritization: a school-based cross-sectional study in Wuhai, Inner Mongolia, China

**DOI:** 10.3389/fpubh.2026.1923480

**Published:** 2026-09-29

**Authors:** Hongyan Wang, Xiafei Liu, Biao Wang, Lijuan Duan, Xia Zhang, Ying Li, Cuitao Yan, Donghu Hai

**Affiliations:** Wuhai Hospital of Traditional Chinese and Mongolian Medicine, Wuhai, Inner Mongolia Autonomous Region, China

**Keywords:** adolescents, children, machine learning, risk prioritization, school health, scoliosis screening

## Abstract

**Background:**

Questionnaire-based prescreening is inexpensive, but its ability to identify students with abnormal scoliosis screening findings is uncertain. We assessed whether questionnaire variables improved prediction beyond a demographic and anthropometric baseline and whether simple on-site physical signs could further improve risk prioritization. The outcome was initial screening positivity, defined by a positive Adams forward-bend test together with an angle of trunk rotation (ATR) of ≥5°.

**Methods:**

This school-based cross-sectional study included 3,559 students aged 10–18 years from four schools in Wuhai, Inner Mongolia, China. Participants underwent anthropometry, postural examination, the Adams test, electronic ATR measurement after a visually positive Adams test, and a structured questionnaire. Questionnaire models were assessed first. As questionnaire variables did not improve the demographic and anthropometric baseline, the final models used age, sex, grade, BMI, shoulder asymmetry, and foot arch morphology. Logistic regression, random forest, and XGBoost were evaluated with five-fold stratified cross-validation repeated three times.

**Results:**

Of 3,559 students, 392 (11.01%) met the initial screening-positive definition. Among models without physical signs, the demographic and anthropometric baseline model performed best (ROC-AUC, 0.633; PR-AUC, 0.163), while adding all or selected questionnaire variables did not improve performance. Among physical-sign models, random forest had the highest ROC-AUC (0.787) and PR-AUC (0.400), and the lowest Brier score (0.082), although its ROC-AUC gain over logistic regression was modest (0.014; 95% CI, −0.001 to 0.029). Prioritizing the top 30% by risk identified 258 of 392 positive results (65.8%); approximately 60.4% of students required prioritization to achieve 90% sensitivity.

**Conclusion:**

Questionnaire information did not support individual prescreening. Simple physical signs improved internal risk ranking for initial screening positivity, but the model cannot replace the combined screening procedure or diagnosis. External validation against clinical and radiographic reference standards is needed.

## 1 Introduction

Scoliosis is a three-dimensional deformity of the spine, and adolescent idiopathic scoliosis is the most common type during growth (1, 2). Mild scoliosis may be asymptomatic and may initially present only as shoulder, scapular, waist, or trunk asymmetry. These subtle signs can be overlooked, particularly during periods of rapid growth. School-based screening provides an opportunity to assess large numbers of otherwise asymptomatic students and may support early identification and referral (3–9). Visual postural examination and the Adams forward-bend test are simple and radiation-free methods commonly used for initial screening. Current Chinese guidance includes visual inspection and the forward-bending test as components of school screening and recommends combining screening methods when feasible (10). Quantitative measurement of the angle of trunk rotation (ATR) with a scoliometer can improve objectivity, whereas radiographic Cobb-angle measurement is required to confirm structural scoliosis and assess severity (3, 4). Screening programs must therefore balance early case finding against false-positive referrals, unnecessary imaging, and resource burden (3, 4, 11, 12). Initial screening findings should be interpreted as indicators for further evaluation rather than as a diagnosis.

The epidemiological characteristics of scoliosis screening vary by region, age, population, and outcome definition (13–16). Recent Chinese school studies have reported positive screening rates of 3.9% in Zhejiang, 2.37% in Gannan, and 2.23% in Hangzhou (17–19). Age, sex, body size, and growth-related characteristics have been associated with scoliosis screening results, although findings vary across populations (18, 20–23). School-related and lifestyle factors, including reading and writing posture, sedentary behavior, sleep, physical activity, and schoolbag-related characteristics, have also been examined (19, 20, 24–26). Some studies have reported associations between abnormal screening findings and unfavorable posture, lower physical activity, or body habitus, whereas others have reported inconsistent results. Such differences may reflect variation in study populations, outcome definitions, and self-reported measurements. Importantly, most available evidence is cross-sectional and does not establish that these behavioral factors cause structural scoliosis.

Most previous studies have focused on group-level associations rather than whether demographic and questionnaire information can discriminate individual students with abnormal screening findings (17, 20, 23). A statistically significant group difference does not necessarily provide sufficient information for individual risk identification. Questionnaires are inexpensive and scalable in large school populations, but self-reported behaviors are indirect and may be affected by recall, reporting, and temporal variability. Recent school health screening programs also emphasize that initial screening should be linked to further standardized assessment and referral rather than treated as a stand-alone diagnosis (27). In contrast, directly observed postural signs may provide more immediate information about visible body asymmetry (24, 28). Determining whether questionnaire variables add predictive information beyond basic demographic and anthropometric characteristics, and whether simple on-site physical signs provide additional value for risk prioritization, may therefore be useful in school settings with limited assessment capacity.

Machine-learning methods provide a way to integrate multiple characteristics and estimate individual-level risk. Previous studies have applied machine learning or artificial intelligence to scoliosis-related prediction and screening (29–33). However, many existing models were developed using hospital populations, radiographs, standardized images, or specialized equipment, which may limit their direct application to routine school settings. In addition, relatively few studies have examined whether questionnaire variables improve prediction beyond basic demographic and anthropometric information or whether simple on-site physical signs provide additional value. Model performance alone also does not determine practical usefulness. In school screening, the proportion of students prioritized for further evaluation must be considered together with the proportion of screening-positive students identified or missed.

To address these gaps, we conducted a school-based cross-sectional study in four schools in Wuhai, Inner Mongolia, China. We examined whether questionnaire variables improved prediction beyond a demographic and anthropometric baseline and whether simple on-site physical signs further improved risk ranking for standardized evaluation. We hypothesized that questionnaire variables would add limited discrimination, whereas observed physical signs would provide additional predictive information. The models predicted the study-defined initial screening outcome, defined by a visually positive Adams forward-bend test together with ATR ≥ 5°.

## 2 Methods

### 2.1 Study design and participants

This school-based cross-sectional study was conducted in Wuhai, Inner Mongolia Autonomous Region, China, from September to December 2024. Students were recruited by cluster sampling from four primary, middle, and high schools. All eligible students with completed consent procedures were invited to participate. The field assessment included anthropometric measurements, visual postural examination, the Adams forward-bend test, electronic ATR measurement for visually Adams-positive students, and a structured questionnaire. The study aimed to estimate the initial screening-positive rate and to evaluate internally validated models for risk prioritization; it was not designed to estimate radiographically confirmed scoliosis prevalence.

Students were eligible if they were 10–18 years old, were enrolled in a participating school, could stand independently in a natural posture, and could complete the Adams forward bend test, anthropometric measurements, and core questionnaire items. Students were excluded if pain or limited mobility prevented correct performance of the test; data for the primary outcome or key predictors were missing; or they had a known congenital spinal deformity, neuromuscular scoliosis, skeletal dysplasia, or another secondary or pathological cause of scoliosis. Records with unresolved logical inconsistencies were also excluded. Because cluster sampling was used, the final sample consisted of all eligible students who completed the core assessments. Model development and internal validation were reported with reference to the TRIPOD+AI recommendations (34).

### 2.2 Ethical clearance

The study was conducted in accordance with the Declaration of Helsinki. The study protocol and consent procedures were reviewed and approved by the Medical Ethics Committee of Wuhai Hospital of Traditional Chinese and Mongolian Medicine, Wuhai, China, on 28 August 2024. Administrative approval was also obtained from the local education authorities before fieldwork.

All participants and their legal guardians were provided with comprehensive details regarding the study objectives, methodology, and data privacy safeguards. Written informed consent was secured from the parents or legal guardians of all minors, while written assent was additionally obtained from participants aged 13 years and older. Every participant retained the right to unconditionally withdraw from the study at any time. To guarantee privacy and confidentiality, all collected data were rigorously de-identified before entry and subsequent analysis.

### 2.3 Screening procedures and outcome definition

Screening procedures were based on the Chinese national standard for spinal curvature screening in children and adolescents (35) and were conducted by trained clinicians and public health staff. Students removed their shoes and stood naturally with their feet together, knees extended, and arms at their sides. Examiners assessed shoulder level, scapular prominence, waistline symmetry, trunk shift, and pelvic tilt. Shoulder asymmetry was defined as a visible difference in acromion height while standing. These postural findings were recorded as part of the standardized physical examination and were not used to define the primary outcome. Shoulder asymmetry and foot arch morphology were the on-site physical-sign variables included in the prediction models.

Examiners then performed the Adams forward-bend test. Students kept their knees extended and feet together and bent forward until the back was approximately parallel to the floor. Examiners visually inspected the thoracic, thoracolumbar, and lumbar regions from behind. A visually positive test was defined by a clear unilateral rib or lumbar prominence or trunk-height asymmetry. Students with a visually positive Adams test then underwent quantitative ATR measurement using an electronic angle-of-trunk-rotation (ATR) measuring device (Fangtian Huaji®, Green Rainbow Prosthetics and Orthotics Technology Co., Ltd., Beijing, China), according to the manufacturer’s standardized procedure. Under the prespecified field protocol, initial screening positivity required both a visually positive Adams test and an ATR of ≥5°. This was an initial screening definition for suspected scoliosis, not a radiographic diagnosis or a validated diagnostic reference standard.

### 2.4 Data collection and candidate predictors

Candidate predictors covered four domains: demographic characteristics, anthropometric measures, directly observed on-site physical signs, and questionnaire variables. Variables were chosen from previous school-based studies and the national screening standard (20, 35, 36). The questionnaire variables were evaluated as exploratory self-reported characteristics rather than established diagnostic indicators. Before data collection, experts reviewed the questionnaire for content and clarity, and a pilot study evaluated comprehension, completion time, and feasibility.

Height, weight, and sitting height were measured with calibrated anthropometric equipment. Students wore light school uniforms and removed shoes, headwear, and heavy outerwear. Height and weight were recorded to the nearest 0.1 cm and 0.1 kg, respectively. BMI was calculated as weight in kilograms divided by height in meters squared. Sitting height was measured with the student seated upright, the ischial tuberosities against the backboard, and the head in the Frankfort horizontal plane. The standing-height-to-sitting-height ratio was then calculated.

Foot arch morphology was assessed during a natural weight-bearing stance and classified as normal, flat, or high based on the medial longitudinal arch. A flat foot was defined by a depressed or absent arch and increased midfoot contact with the floor. A high arch was defined by an elevated arch and reduced midfoot contact. Shoulder asymmetry was assessed visually from the difference in acromion height.

The questionnaire was adapted from previous school-based studies and the national standard (20, 35) and was modified for local students. The final version was established after expert review and pilot testing in 80 students. Students completed the questionnaire in the classroom under investigator guidance, and completed forms were checked immediately. Because these variables were self-reported, they were treated as exploratory candidate predictors and not as substitutes for physical screening.

The questionnaire included school and backpack characteristics; reading, writing, and posture habits; sleep and physical activity; and health and family-history items. The complete set of questionnaire variables and their group distributions is provided in Supplementary Table S1.

### 2.5 Quality control

All screening staff completed standardized training led by public health and clinical personnel. Measurement equipment was calibrated before use. Students completed the questionnaire under the supervision of teachers and the research team. Data were entered independently by two staff members, and discrepancies were resolved against the original forms. Records with extensive missing data or unresolved inconsistencies were excluded.

### 2.6 Modeling and evaluation

All prediction analyses used five-fold stratified cross-validation repeated three times. Preprocessing and model fitting were performed separately within each training fold, and each participant received out-of-fold predictions that were averaged across the three repeats. The modeling strategy addressed two analytical questions and did not represent a sequential screening pathway implemented during field data collection. All 3,559 eligible participant records were retained; identical covariate profiles were not automatically treated as duplicate participants in the absence of a documented duplicate identifier.

The first analysis assessed the incremental predictive value of questionnaire variables beyond the base model. The base model included age, sex, grade, and BMI. The full questionnaire model added all 17 lifestyle and questionnaire variables. The reduced questionnaire model used Elastic Net within each training fold to rank questionnaire variables and retained up to five items before logistic regression. This nested selection prevented information from the validation fold entering variable selection.

The second analysis evaluated post-assessment risk prioritization after simple physical signs were available. Because neither the full nor the reduced questionnaire model improved on the base model, questionnaire variables were not carried into the final physical-sign-enhanced models. Age, sex, grade, BMI, shoulder asymmetry, and foot arch morphology were entered into logistic regression, random forest, and XGBoost models. For the random forest model, variable importance was estimated by permutation on held-out validation folds and expressed as the decrease in ROC-AUC after permutation.

Model performance was summarized by ROC-AUC, precision-recall AUC (PR-AUC), Brier score, calibration plots, and repeated-fold ROC-AUC (37, 38). Ninety-five percent confidence intervals were estimated by 1,000 bootstrap resamples of aggregated out-of-fold predictions. Paired bootstrap ROC-AUC differences compared the classifiers. Physical-sign models were compared with the base model, the best model without on-site physical signs.

Decision curve analysis estimated decision-analytic net benefit across threshold probabilities from 0.01 to 0.40 (39). The comparison strategies were prioritizing all students and prioritizing none. Risk-ranking analyses then quantified how many initial screen-positive students would be identified if a specified proportion of students were prioritized for further standardized evaluation. Additional thresholds were selected to achieve approximately 80, 90, and 95% sensitivity. These analyses were simulated downstream prioritization scenarios based on internal validation; they do not demonstrate that the model can replace the combined initial screening procedure, reduce the need for trained screening personnel, or diagnose scoliosis.

### 2.7 Statistical analysis

All analyses were conducted in Python using scikit-learn and XGBoost. Statistical tests were two-sided, and *p* < 0.05 was considered statistically significant. Continuous and categorical missing values were imputed using the median and most frequent category, respectively, within training folds only. Standardization, one-hot encoding, and Elastic Net selection were also restricted to training folds to prevent information leakage. Model reporting was revised with reference to TRIPOD+AI recommendations (34).

Normally distributed continuous variables are presented as mean (standard deviation) and were compared using Welch’s t test. Skewed continuous variables are presented as median [interquartile range] and were compared using the Mann–Whitney U test. Categorical variables are presented as n (%) and were compared using Pearson’s chi-square test or Fisher’s exact test, as appropriate. Group comparisons were descriptive and exploratory; *p* values were not adjusted for multiple testing and should not be interpreted as evidence of causality. Core characteristics are shown in Table 1, and the complete descriptive table is provided as Supplementary Table S1.

**Table 1:** Baseline characteristics of the study participants.

Characteristics	Total sample (*n* = 3,559)	Initial screening status		*p*
		Negative (*n* = 3,167)	Positive (*n* = 392)	
Age, years	14.34 (1.93)	14.27 (1.94)	14.94 (1.75)	<0.001*
Height-to-sitting-height ratio	1.88 [1.84, 1.92]	1.88 [1.84, 1.92]	1.89 [1.85, 1.93]	<0.001*
Height, cm	165.16 (9.81)	164.81 (9.85)	168.03 (8.96)	<0.001*
Weight, kg	61.19 (15.76)	61.20 (15.92)	61.15 (14.47)	0.955
BMI, kg/m^2^	22.22 (4.45)	22.31 (4.49)	21.51 (4.02)	<0.001*
Sex				0.062
Female	1,874 (52.66)	1,685 (53.20)	189 (48.21)	
Male	1,685 (47.34)	1,482 (46.80)	203 (51.79)	
Grade				<0.001*
Primary school	515 (14.47)	487 (15.38)	28 (7.14)	
Middle school	1,628 (45.74)	1,475 (46.57)	153 (39.03)	
High school	1,416 (39.79)	1,205 (38.05)	211 (53.83)	
Shoulder asymmetry				<0.001*
No	3,053 (85.78)	2,866 (90.50)	187 (47.70)	
Yes	506 (14.22)	301 (9.50)	205 (52.30)	
Foot arch morphology				<0.001*
Normal	3,425 (96.23)	3,067 (96.84)	358 (91.33)	
Flat foot	131 (3.68)	97 (3.06)	34 (8.67)	
High arch	3 (0.08)	3 (0.09)	0 (0.00)	
Average daily sleep duration				<0.001*
< 7 h	2,079 (58.42)	1,809 (57.12)	270 (68.88)	
7–8 h	780 (21.92)	699 (22.07)	81 (20.66)	
> 8 h	700 (19.67)	659 (20.81)	41 (10.46)	
Reading and writing posture				<0.001*
Straight back	1,333 (37.45)	1,217 (38.43)	116 (29.59)	
Hunched back	2,048 (57.54)	1,786 (56.39)	262 (66.84)	
No fixed posture	178 (5.00)	164 (5.18)	14 (3.57)	
Daily sedentary time				0.224
< 2 h	860 (24.16)	775 (24.47)	85 (21.68)	
≥ 2 h	2,699 (75.84)	2,392 (75.53)	307 (78.32)	
PE classes per week				<0.001*
0–2 classes	1,275 (35.82)	1,095 (34.58)	180 (45.92)	
3–4 classes	2,185 (61.39)	1,983 (62.61)	202 (51.53)	
≥ 5 classes	99 (2.78)	89 (2.81)	10 (2.55)	
Family history of spinal issues				0.030*
No	2,072 (58.22)	1,868 (58.98)	204 (52.04)	
Yes	184 (5.17)	162 (5.12)	22 (5.61)	
Uncertain	1,303 (36.61)	1,137 (35.90)	166 (42.35)	

Data are mean (SD), median [IQR], or *n* (%). *p < 0.05. BMI, body mass index; PE, physical education.

## 3 Results

### 3.1 Baseline characteristics of study participants

The study included 3,559 children and adolescents; 3,167 had a negative initial screening result and 392 met the initial screening-positive definition, corresponding to 11.01%. Core characteristics are shown in Table 1 and the complete descriptive results in Supplementary Table S1. Students with initial screening positivity were older (14.94 vs. 14.27 years, *p* < 0.001), taller (168.03 vs. 164.81 cm, *p* < 0.001), and had lower BMI (21.51 vs. 22.31 kg/m^2^, *p* < 0.001). Grade distribution also differed between groups (*p* < 0.001).

Shoulder asymmetry was observed in 52.30% of initial screen-positive students compared with 9.50% of screen-negative students (*p* < 0.001). Flat-foot morphology was also more frequent in the positive group (8.67% vs. 3.06%, *p* < 0.001). Family-history distribution differed between groups (*p* = 0.030). These findings describe associations with the combined initial-screening outcome and should not be interpreted as diagnostic accuracy or causal effects.

Several self-reported characteristics differed between groups. Screen-positive students more often reported sleep shorter than 7 h, hunched reading/writing posture, infrequent desk and chair adjustment, and fewer physical education classes (all *p* ≤ 0.003). Longer after-school writing time and more frequent screen use while lying down also differed between groups (*p* = 0.004 and *p* = 0.006, respectively). Because these were exploratory self-reported variables and multiple comparisons were performed, the findings were used for description rather than causal inference.

No statistically significant differences were observed for myopia, backpack carrying method, daily sedentary time, days of moderate-to-vigorous physical activity, or dietary regularity (all *p* > 0.05).

### 3.2 Questionnaire models

The base model, containing age, sex, grade, and BMI, achieved an ROC-AUC of 0.633 (95% CI, 0.605–0.659), a PR-AUC of 0.163 (95% CI, 0.144–0.191), and a Brier score of 0.096 (Table 2 and Figure 1). Adding all questionnaire variables did not improve discrimination (ROC-AUC, 0.613; PR-AUC, 0.151). The reduced questionnaire model also did not improve performance (ROC-AUC, 0.625; PR-AUC, 0.159). It retained five questionnaire variables in each training fold. Sleep duration and family history were selected in all 15 folds, followed by desk/chair adjustment in 10 folds and after-school writing duration in 9 folds (Supplementary Table S2). These results indicate limited incremental predictive value of questionnaire variables for individual-level identification of initial screening positivity.

**Table 2:** Questionnaire models.

Model	Description	Variables (n)	ROC-AUC (95% CI)	PR-AUC (95% CI)	Brier score
Base	Age, sex, grade, BMI	4	0.633 (0.605–0.659)	0.163 (0.144–0.191)	0.096
Questionnaire	Base + all questionnaire variables	21	0.613 (0.583–0.640)	0.151 (0.133–0.176)	0.097
Reduced questionnaire	Base + ≤ 5 selected variables/fold	4 + 5	0.625 (0.595–0.652)	0.159 (0.140–0.186)	0.096

ROC-AUC, receiver operating characteristic area under the curve; PR-AUC, precision-recall area under the curve; CI, confidence interval; BMI, body mass index. Estimates use repeated cross-validation.

**Figure 1:**
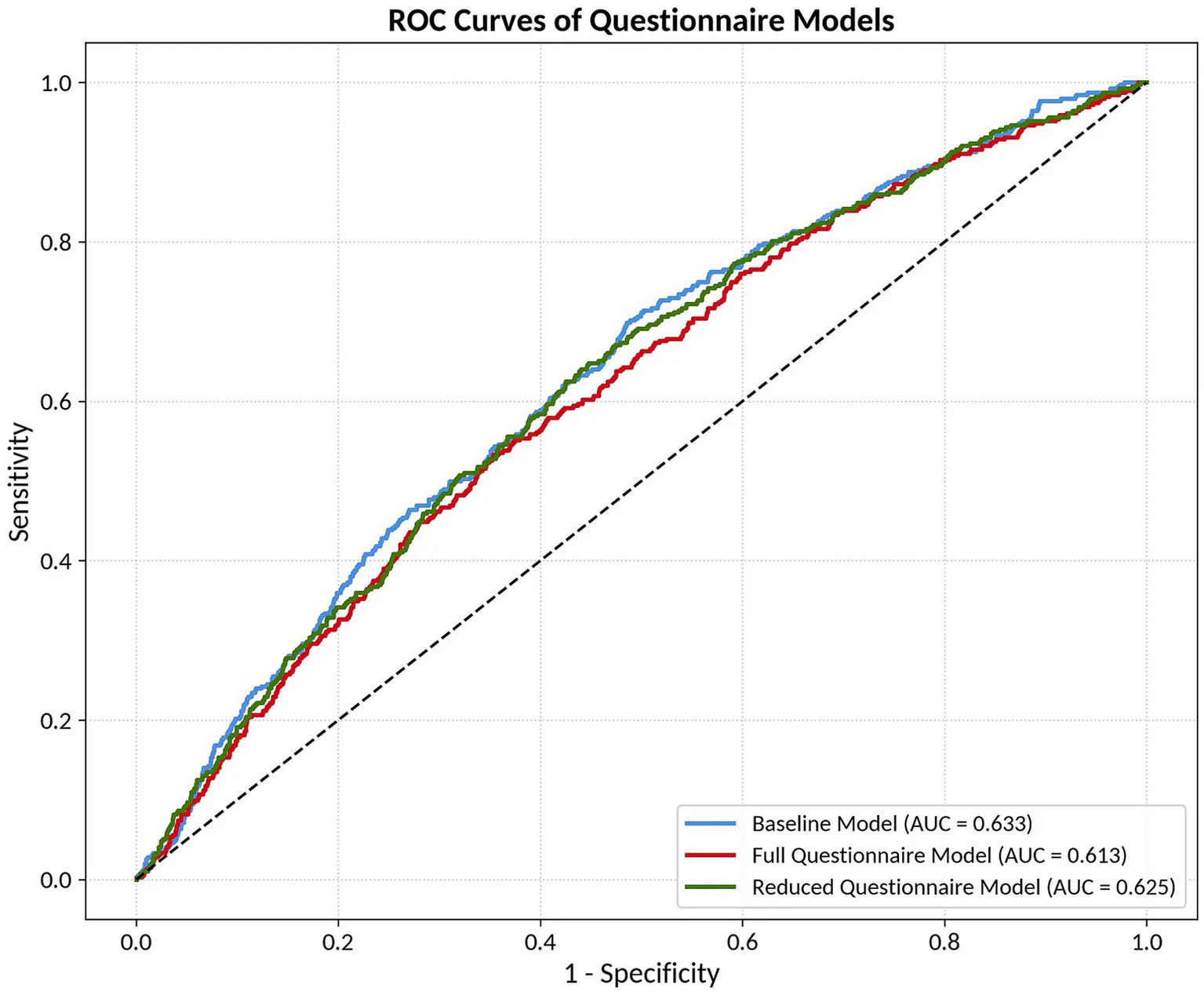
ROC curves for questionnaire models. The base model included age, sex, grade, and BMI; the full model added all questionnaire variables; and the reduced model used up to five variables selected within each training fold.

### 3.3 Physical-sign models

Because questionnaire variables did not improve the demographic and anthropometric baseline, the final physical-sign-enhanced models used six variables: age, sex, grade, BMI, shoulder asymmetry, and foot arch morphology. Random forest had the numerically highest overall performance, with an ROC-AUC of 0.787 (95% CI, 0.762–0.812), PR-AUC of 0.400 (95% CI, 0.354–0.454), and Brier score of 0.082 (95% CI, 0.075–0.089) (Table 3). Logistic regression achieved an ROC-AUC of 0.773, and XGBoost achieved 0.775. Relative to the base model, the absolute ROC-AUC improvements were 0.140 for logistic regression, 0.155 for random forest, and 0.143 for XGBoost.

**Table 3:** Physical-sign models.

Model	ROC-AUC (95% CI)	ΔROC-AUC vs. base model	PR-AUC (95% CI)	Fold ROC-AUC (mean ± SD)	Brier score (95% CI)
Logistic regression	0.773 (0.748–0.798)	+0.140	0.316 (0.278–0.363)	0.775 ± 0.027	0.084 (0.078–0.092)
Random forest	0.787 (0.762–0.812)	+0.155	0.400 (0.354–0.454)	0.789 ± 0.025	0.082 (0.075–0.089)
XGBoost	0.775 (0.750–0.800)	+0.143	0.384 (0.338–0.436)	0.772 ± 0.024	0.083 (0.076–0.090)

All models included age, sex, grade, BMI, shoulder asymmetry, and foot arch morphology. ΔROC-AUC is the difference from the base model (0.633). CIs were estimated by 1,000 bootstraps.

Figure 2A shows the ROC curves. The paired bootstrap ROC-AUC difference between random forest and logistic regression was 0.014 (95% CI, −0.001 to 0.029), indicating that the random forest advantage over the simpler logistic model was modest. The random forest exceeded XGBoost by 0.012 (95% CI, 0.004–0.020). Validation-set permutation importance showed that shoulder asymmetry produced the largest decrease in ROC-AUC when permuted, followed by grade and age (Figure 2B). This importance reflects predictive contribution to initial screening positivity and should not be interpreted as a causal or diagnostic effect.

**Figure 2:**
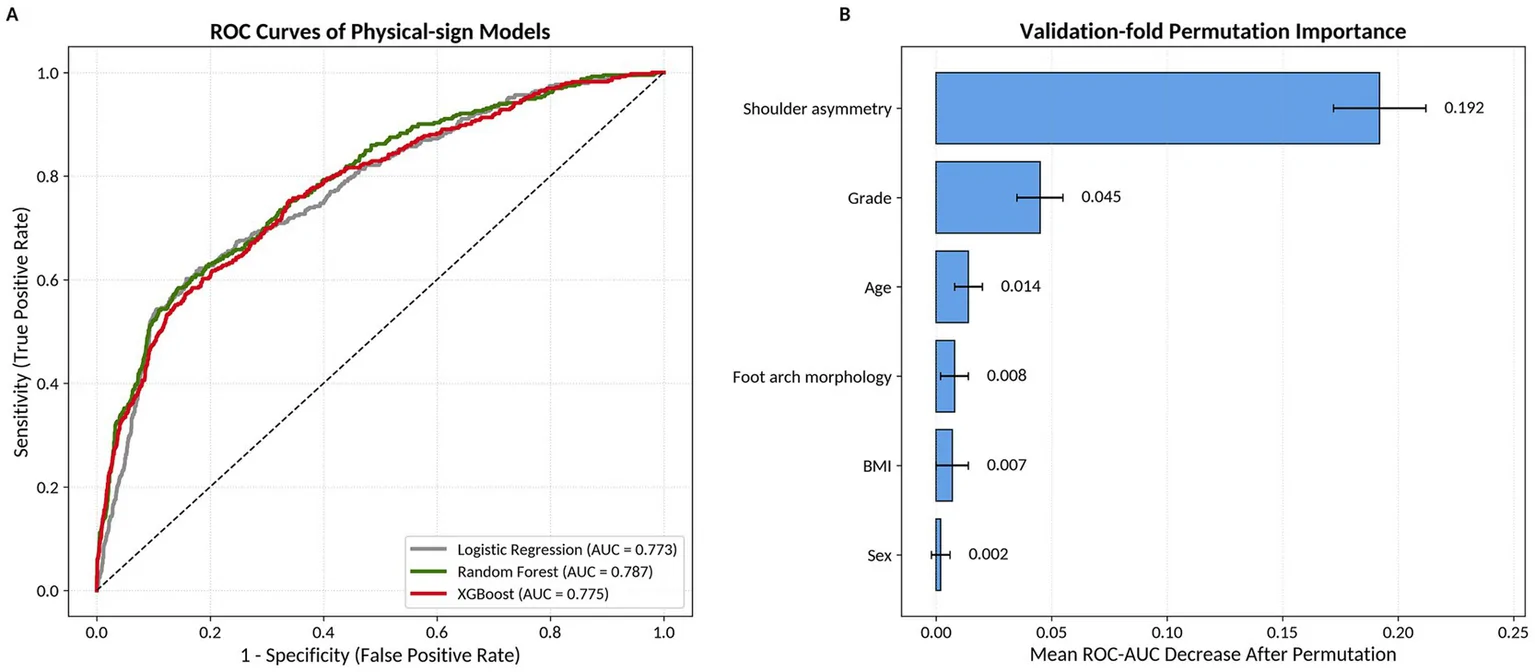
Physical-sign models and predictor importance. **(A)** ROC curves for logistic regression, random forest, and XGBoost. All models included age, sex, grade, BMI, shoulder asymmetry, and foot arch morphology. **(B)** Validation-set permutation importance for random forest; error bars show between-fold SD.

### 3.4 Risk-prioritization scenarios

The random forest calibration plot showed broadly similar predicted and observed initial screen-positive proportions across the observed risk range (Figure 3A). Decision curve analysis showed higher net benefit for the risk model than prioritizing all or prioritizing none across threshold probabilities from 0.01 to 0.40 (Figure 3B). These results are based exclusively on internal out-of-fold predictions and describe decision-analytic utility for the study outcome, not clinical benefit for radiographically confirmed scoliosis.

**Figure 3:**
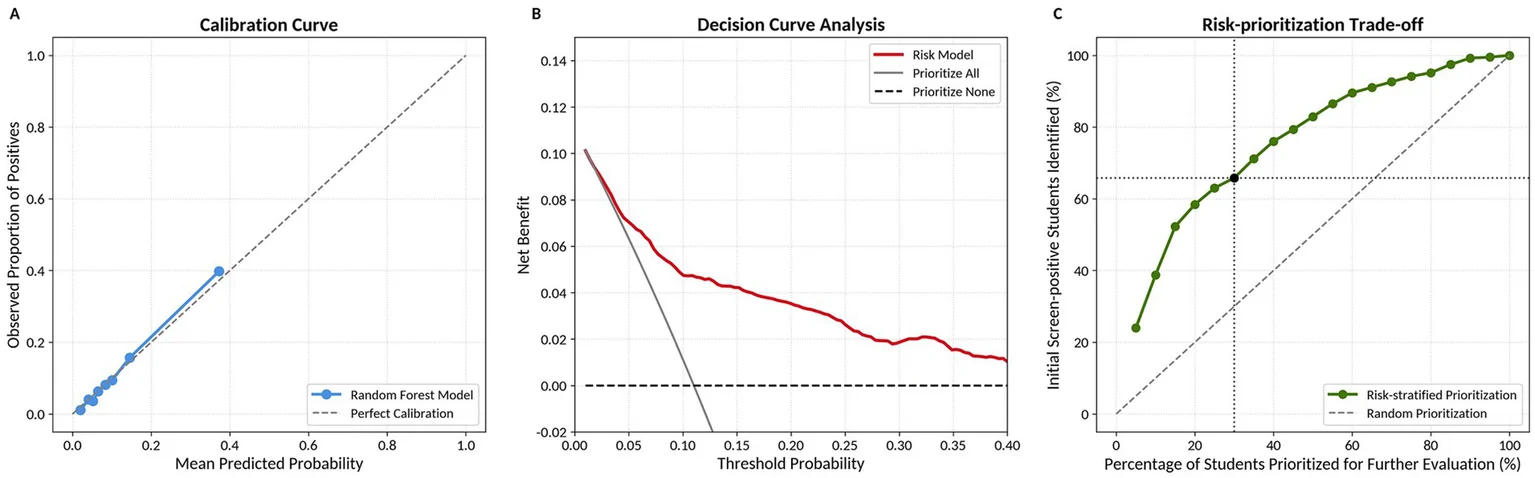
Internal validation and risk prioritization. **(A)** Calibration. **(B)** Decision-curve analysis. **(C)** The proportion of initial screen-positive students identified as the prioritized proportion increased. The top 30% identified 65.8% of positive students. These results concern the combined initial screening outcome, not diagnostic accuracy for structural scoliosis.

At the maximum Youden-index cutoff (0.125), 684 students (19.2%) were prioritized, identifying 229 of 392 initial screen-positive students (58.4% sensitivity; 85.6% specificity). More importantly for a screening context, achieving approximately 80, 90, and 95% sensitivity required prioritizing 45.9, 60.4, and 79.3% of all students, respectively (Table 4). Thus, high sensitivity substantially reduced the apparent workload-saving advantage.

**Table 4:** Risk-prioritization scenarios.

Prioritization strategy	Cutoff	Screen-positive identified, *n*	Missed screen-positive, n	Sensitivity, %	Specificity, %	PPV, %
Maximum Youden index	> 0.125	229	163	58.4	85.6	33.5
Top 30% by predicted risk	> 0.102	258	134	65.8	74.4	24.2
Top 40% by predicted risk	> 0.089	298	94	76.0	64.4	20.9
Approximately 80% sensitivity	> 0.080	314	78	80.1	58.4	19.2
Approximately 90% sensitivity	> 0.060	353	39	90.1	43.3	16.4
Approximately 95% sensitivity	> 0.043	373	19	95.2	22.6	13.2

PPV, positive predictive value. Values are based on repeated cross-validation. Prioritized students were selected for further standardized evaluation after basic on-site assessment.

Risk ranking produced a continuous trade-off between the proportion prioritized and the proportion of initial screen-positive students identified (Figure 3C). Prioritizing the top 30% identified 258 of 392 positive results (65.8%), while prioritizing the top 40% identified 298 (76.0%). These scenarios refer only to prioritization for further standardized evaluation after basic on-site information is available; they should not be interpreted as a replacement for the combined school screening procedure. Additional prioritization scenarios, including the default 0.50 probability cutoff and top-20% prioritization, are provided in Supplementary Table S3.

## 4 Discussion

This study examined 3,559 children and adolescents and addressed two related questions in school scoliosis screening: whether questionnaire information improves identification of initial screening positivity and whether directly observed physical signs add value for risk prioritization. Adding questionnaire variables to the demographic and anthropometric baseline did not improve individual-level discrimination, despite several group-level associations. In contrast, adding simple on-site physical signs substantially improved prediction of the combined initial-screening outcome. Random forest showed the best numerical performance, although its advantage over logistic regression was modest. These findings suggest that directly observed postural information may be more useful for risk ranking than self-reported lifestyle information. However, the prediction target was an initial screening result defined by the Adams test and electronic ATR measurement, rather than radiographically confirmed structural scoliosis. The model should therefore be interpreted as an exploratory risk-ranking tool rather than an alternative screening or diagnostic protocol.

The 11.01% initial screening-positive rate was higher than rates reported in several recent regional studies (17–19, 23). Differences in age distribution, sampling, screening procedures, and outcome definitions may contribute to this variation. In the present study, initial screening positivity required both a visually positive Adams forward-bend test and ATR ≥ 5°. Quantitative assessment of trunk rotation can improve the objectivity of visual forward-bend screening (40), although our procedure did not include radiographic confirmation. The observed rate should therefore be interpreted as initial screening positivity for suspected scoliosis rather than prevalence of adolescent idiopathic scoliosis. Screening-positive students were older, taller, and had lower BMI, and shoulder asymmetry and flat-foot morphology were more frequent in this group. Previous studies have also linked age, growth-related characteristics, and body size with scoliosis screening findings, although results vary across populations (18, 20–23). Body habitus may also affect trunk-rotation measurements, as previous work has shown variation in scoliosis-related surface measurements with BMI (41).

The questionnaire analysis provides an important negative finding. Several self-reported lifestyle characteristics differed between groups, but neither the full questionnaire model nor Elastic Net-based reduction improved discrimination beyond the demographic and anthropometric baseline. This shows that group-level statistical associations do not necessarily translate into useful individual-level prediction. Self-reported behaviors may be affected by recall error, reporting bias, temporal variation, and correlations among questionnaire items. Previous findings on posture, physical activity, sleep, and scoliosis-related outcomes have also been inconsistent (19, 20, 24–26). These questionnaire findings should therefore be considered descriptive and hypothesis-generating rather than causal or clinically predictive.

In contrast, simple observed physical signs improved model performance. Shoulder asymmetry showed the largest predictive contribution, which is clinically plausible because it directly reflects visible postural asymmetry. Foot arch morphology contributed less and should be regarded as supplementary rather than diagnostic. Previous machine-learning studies have often relied on radiographs, standardized images, hospital-based variables, or specialized equipment (29–33), which may limit direct use in routine school settings. In the present study, the predictive information came mainly from simple on-site variables. Random forest achieved the highest ROC-AUC and PR-AUC and the lowest Brier score, but its ROC-AUC advantage over logistic regression was small. This suggests that much of the useful information was captured by a limited number of interpretable variables rather than by complex nonlinear modeling alone.

The risk-prioritization analysis further clarifies the model’s practical value. Prioritizing the top 30% of students identified 65.8% of initial screen-positive students, whereas approximately 60% had to be prioritized to reach 90% sensitivity. Thus, higher sensitivity substantially reduced the apparent workload-saving advantage. Decision curve analysis nevertheless showed higher net benefit for the model than prioritizing all or none across the evaluated threshold range. These findings suggest that risk ranking may help determine the order of further standardized evaluation when screening capacity is limited. However, these scenarios were based only on internal validation and do not show that the model can reduce costs, replace the combined screening procedure, or improve clinical outcomes.

Several limitations should be considered. First, the cross-sectional design does not permit causal inference. Second, the screening outcome was not confirmed radiographically, and the electronic ATR device was not independently validated against a radiographic reference standard in this study. Because ATR was measured only after a visually positive Adams test, subtle rotational deformity may also have been missed. Third, shoulder asymmetry is itself a visible sign related to the screening process, which limits interpretation of the model as an independent diagnostic method. Fourth, questionnaire variables were self-reported, and repeatability of the on-site physical assessments was not formally quantified. Fifth, participants came from four schools in one city, and only internal repeated cross-validation was performed. Because cross-validation was conducted at the participant level rather than grouped by school, performance may be optimistic when applied to entirely new schools. External validation across regions, schools, and screening teams is therefore required.

## 5 Conclusion

In conclusion, adding questionnaire information to the demographic and anthropometric baseline did not improve discrimination of initial screening positivity, whereas adding simple on-site physical signs improved internal risk ranking. Random forest showed the best numerical performance. However, the model was developed for risk prioritization rather than diagnosis and should not replace the combined screening procedure or trained clinical assessment. External prospective validation against clinical and radiographic reference standards is required before routine use.

## Data Availability

The datasets presented in this article are not publicly available because they contain data from minors and access is restricted to protect participant privacy. Requests to access the datasets should be directed to the corresponding author and will be considered subject to approval by the relevant ethics committee and applicable regulations.
