# Supplementary material for: Initial screening positivity for suspected scoliosis and machine learning-assisted risk prioritization: a school-based cross-sectional study in Wuhai, Inner Mongolia, China
Source: Front Public Health. 2026 Sep 29;14:1923480. doi: 10.3389/fpubh.2026.1923480 (PMC13658430; doi:10.3389/fpubh.2026.1923480)
Supplement: Supplementary file 1 [file Table_1.DOCX]

Supplementary Table S1. Participant characteristics

| Characteristic | Total (n = 3,559) | Negative (n = 3,167) | Positive (n = 392) | p |
| --- | --- | --- | --- | --- |
| Age, years | 14.34 (1.93) | 14.27 (1.94) | 14.94 (1.75) | <0.001* |
| Height-to-sitting-height ratio | 1.88 [1.84, 1.92] | 1.88 [1.84, 1.92] | 1.89 [1.85, 1.93] | <0.001* |
| Height, cm | 165.16 (9.81) | 164.81 (9.85) | 168.03 (8.96) | <0.001* |
| Weight, kg | 61.19 (15.76) | 61.20 (15.92) | 61.15 (14.47) | 0.955 |
| BMI, kg/m² | 22.22 (4.45) | 22.31 (4.49) | 21.51 (4.02) | <0.001* |
| Sex |  |  |  | 0.062 |
| Female | 1874 (52.66) | 1685 (53.20) | 189 (48.21) |  |
| Male | 1685 (47.34) | 1482 (46.80) | 203 (51.79) |  |
| Grade |  |  |  | <0.001* |
| Primary school | 515 (14.47) | 487 (15.38) | 28 (7.14) |  |
| Middle school | 1628 (45.74) | 1475 (46.57) | 153 (39.03) |  |
| High school | 1416 (39.79) | 1205 (38.05) | 211 (53.83) |  |
| Shoulder asymmetry |  |  |  | <0.001* |
| No | 3053 (85.78) | 2866 (90.50) | 187 (47.70) |  |
| Yes | 506 (14.22) | 301 (9.50) | 205 (52.30) |  |
| Foot arch morphology |  |  |  | <0.001* |
| Normal | 3425 (96.23) | 3067 (96.84) | 358 (91.33) |  |
| Flat foot | 131 (3.68) | 97 (3.06) | 34 (8.67) |  |
| High arch | 3 (0.08) | 3 (0.09) | 0 (0.00) |  |
| Myopia |  |  |  | 0.071 |
| No | 1057 (29.70) | 956 (30.19) | 101 (25.77) |  |
| Yes | 2502 (70.30) | 2211 (69.81) | 291 (74.23) |  |
| Backpack carrying method |  |  |  | 0.37 |
| Single shoulder | 118 (3.32) | 108 (3.41) | 10 (2.55) |  |
| Double shoulder | 3441 (96.68) | 3059 (96.59) | 382 (97.45) |  |
| Postural self-monitoring |  |  |  | <0.001* |
| Indifferent or occasional | 1912 (53.72) | 1670 (52.73) | 242 (61.73) |  |
| Frequent or constant | 1647 (46.28) | 1497 (47.27) | 150 (38.27) |  |
| Average daily sleep duration |  |  |  | <0.001* |
| < 7 h | 2079 (58.42) | 1809 (57.12) | 270 (68.88) |  |
| 7–8 h | 780 (21.92) | 699 (22.07) | 81 (20.66) |  |
| > 8 h | 700 (19.67) | 659 (20.81) | 41 (10.46) |  |
| Sleep posture |  |  |  | 0.058 |
| Supine | 803 (22.56) | 717 (22.64) | 86 (21.94) |  |
| Lateral | 2426 (68.17) | 2144 (67.70) | 282 (71.94) |  |
| Other | 330 (9.27) | 306 (9.66) | 24 (6.12) |  |
| After-school writing duration |  |  |  | 0.004* |
| < 1 h | 601 (16.89) | 557 (17.59) | 44 (11.22) |  |
| 1–3 h | 2532 (71.14) | 2240 (70.73) | 292 (74.49) |  |
| ≥ 3 h | 426 (11.97) | 370 (11.68) | 56 (14.29) |  |
| Chest-to-desk distance > one fist |  |  |  | 0.163 |
| Occasionally or frequently | 2460 (69.12) | 2177 (68.74) | 283 (72.19) |  |
| Always | 1099 (30.88) | 990 (31.26) | 109 (27.81) |  |
| Days of MVPA in past week |  |  |  | 0.067 |
| 0–2 days | 1505 (42.29) | 1318 (41.62) | 187 (47.70) |  |
| 3–5 days | 1676 (47.09) | 1507 (47.58) | 169 (43.11) |  |
| 6–7 days | 378 (10.62) | 342 (10.80) | 36 (9.18) |  |
| Previous bracing or rehabilitation |  |  |  | <0.001* |
| No | 3417 (96.01) | 3061 (96.65) | 356 (90.82) |  |
| Yes | 142 (3.99) | 106 (3.35) | 36 (9.18) |  |
| Lower back discomfort |  |  |  | 0.011* |
| None | 1799 (50.55) | 1610 (50.84) | 189 (48.21) |  |
| Occasional | 1523 (42.79) | 1360 (42.94) | 163 (41.58) |  |
| Frequent | 237 (6.66) | 197 (6.22) | 40 (10.20) |  |
| Reading and writing posture |  |  |  | <0.001* |
| Straight back | 1333 (37.45) | 1217 (38.43) | 116 (29.59) |  |
| Hunched back | 2048 (57.54) | 1786 (56.39) | 262 (66.84) |  |
| No fixed posture | 178 (5.00) | 164 (5.18) | 14 (3.57) |  |
| Daily sedentary time |  |  |  | 0.224 |
| < 2 h | 860 (24.16) | 775 (24.47) | 85 (21.68) |  |
| ≥ 2 h | 2699 (75.84) | 2392 (75.53) | 307 (78.32) |  |
| Class seat rotation frequency |  |  |  | 0.143 |
| Never or once per semester | 291 (8.18) | 261 (8.24) | 30 (7.65) |  |
| 1–2 times per month | 1230 (34.56) | 1077 (34.01) | 153 (39.03) |  |
| Once per week | 2038 (57.26) | 1829 (57.75) | 209 (53.32) |  |
| Desk and chair adjustment |  |  |  | 0.003* |
| Never or unadjustable | 2267 (63.70) | 1988 (62.77) | 279 (71.17) |  |
| 1–2 times per month | 768 (21.58) | 707 (22.32) | 61 (15.56) |  |
| Once per week | 524 (14.72) | 472 (14.90) | 52 (13.27) |  |
| PE classes per week |  |  |  | <0.001* |
| 0–2 classes | 1275 (35.82) | 1095 (34.58) | 180 (45.92) |  |
| 3–4 classes | 2185 (61.39) | 1983 (62.61) | 202 (51.53) |  |
| ≥ 5 classes | 99 (2.78) | 89 (2.81) | 10 (2.55) |  |
| Family history of spinal issues |  |  |  | 0.030* |
| No | 2072 (58.22) | 1868 (58.98) | 204 (52.04) |  |
| Yes | 184 (5.17) | 162 (5.12) | 22 (5.61) |  |
| Uncertain | 1303 (36.61) | 1137 (35.90) | 166 (42.35) |  |
| Screen use while lying down |  |  |  | 0.006* |
| Never or occasionally | 2116 (59.45) | 1908 (60.25) | 208 (53.06) |  |
| Frequently or always | 1443 (40.55) | 1259 (39.75) | 184 (46.94) |  |
| Regular diet |  |  |  | 0.07 |
| No | 704 (19.78) | 613 (19.36) | 91 (23.21) |  |
| Yes | 2855 (80.22) | 2554 (80.64) | 301 (76.79) |  |
| Picky eater |  |  |  | 0.153 |
| No | 2613 (73.42) | 2337 (73.79) | 276 (70.41) |  |
| Yes | 946 (26.58) | 830 (26.21) | 116 (29.59) |  |

**Note:** This table reports all collected variables. Group comparisons are exploratory and p values are unadjusted. The 17 prespecified questionnaire predictors used for modeling were myopia, backpack carrying method, postural self-monitoring, average daily sleep duration, sleep posture, after-school writing duration, chest-to-desk distance > one fist, days of MVPA in the past week, reading and writing posture, daily sedentary time, class seat rotation frequency, desk and chair adjustment, PE classes per week, family history of spinal issues, screen use while lying down, regular diet, and picky eater. Previous bracing or rehabilitation and lower back discomfort were collected for descriptive purposes and were not included in the questionnaire-based prediction models.

Supplementary Table S2. Questionnaire-variable selection

| Questionnaire variable | Selected training folds, n / 15 | Selection frequency, % |
| --- | --- | --- |
| Family history of spinal issues | 15 / 15 | 100.0 |
| Average daily sleep duration | 15 / 15 | 100.0 |
| Desk and chair adjustment | 10 / 15 | 66.7 |
| After-school writing duration | 9 / 15 | 60.0 |
| Postural self-monitoring | 7 / 15 | 46.7 |
| Myopia | 4 / 15 | 26.7 |
| Sleep posture | 4 / 15 | 26.7 |
| Reading and writing posture | 4 / 15 | 26.7 |
| Days of MVPA in past week | 2 / 15 | 13.3 |
| Regular diet | 2 / 15 | 13.3 |
| Daily sedentary time | 2 / 15 | 13.3 |
| PE classes per week | 1 / 15 | 6.7 |

**Note:** The reduced model retained up to five questionnaire variables per training fold. Only questionnaire variables selected in at least one of the 15 training folds generated by three repeats of five-fold cross-validation are shown; selection frequencies are descriptive.

Supplementary Table S3. Additional prioritization scenarios

| Strategy | Cutoff | Prioritized, n (%) | Screen-positive identified, n | Missed, n | Sensitivity, % | Specificity, % | PPV, % |
| --- | --- | --- | --- | --- | --- | --- | --- |
| Default probability cutoff 0.50 | > 0.500 | 53 (1.5) | 38 | 354 | 9.7 | 99.5 | 71.7 |
| Top 20% by predicted risk | > 0.122 | 712 (20.0) | 229 | 163 | 58.4 | 84.7 | 32.2 |

**Note:** Screen-positive refers to the study-defined initial screening-positive outcome (visually positive Adams forward-bend test together with ATR ≥5°). Values are based on repeated cross-validation.
